# The Possibility to Use Pine Timber Pieces with Small Size in the Production of Glulam Beams

**DOI:** 10.3390/ma15093154

**Published:** 2022-04-27

**Authors:** Dorota Dziurka, Jakub Kawalerczyk, Joanna Walkiewicz, Adam Derkowski, Radosław Mirski

**Affiliations:** Department of Mechanical Wood Technology, Poznań University of Life Sciences, ul. Wojska Polskiego 28, 60-627 Poznan, Poland; jakub.kawalerczyk@up.poznan.pl (J.K.); joanna.siuda@up.poznan.pl (J.W.); adam.derkowski@up.poznan.pl (A.D.); radoslaw.mirski@up.poznan.pl (R.M.)

**Keywords:** glulam beams, engineered wood products, low-quality timber, pine, structural elements

## Abstract

Engineered wood products, such as glulam beams, attract much attention from the building industry in recent years. Therefore, there is a constant necessity to seek new models of structural beams, which assume the use of outsized sawn wood pieces as an alternative for the standard construction timber. Three variants of glulam beams, composed of the main yield and side boards arranged in various structures, were proposed. Moreover, the usefulness of wedge-jointed, small-sized timber pieces was also investigated. The manufactured beams were tested, in terms of their mechanical properties, such as bending strength, elastic energy, modulus of elasticity, and resilience. The outcomes have shown that the beams manufactured using wedge-bonded timber of lower grade do not deviate considerably from beams produced from homogeneous lamellas. Furthermore, the results of modulus of elasticity, in the case of the three-layered beams composed of both small-sized non-homogenous main yield and side boards, exceeded the requirements from EN 14080. It allowed us to classify the obtained materials as GL 32c, which is the highest grade specified within the standard.

## 1. Introduction

Wood is a material of a natural origin, which has been used for centuries as a construction product. Its constantly growing popularity is due to a many favorable features, when compared with the others, such as steel and concrete [1,2,3]. These synthetic structural materials are usually characterized by exceptional strength properties; however, they suffer from either the complex manufacturing process, negative environmental impact, or high weight [4,5]. Moreover, a growing interest in engineered wood products (EWP) application in buildings results from the increasing attention focused on the sustainable construction industry, aiming at the utilization of natural materials, which may be further re-used [6,7]. Many studies determining the possibility of using a various softwood and hardwood species, originating both from and outside Europe, in glulam beams have been conducted [8,9,10,11,12]. Research show that timber can be used to manufacture construction materials for both high- and low-rise buildings. For example, the EWP have been used as prefabricated construction elements to build a 53 m high residential building at the University of British Columbia in Vancouver, Canada [13].

The fluctuations in morphological and mechanical properties are the major consequence of natural growing process. The morphological characteristics of timber are strongly dependent on the fiber course, which can be significantly disturbed by the presence of wood defects [14]. The quality of Scots pine, commonly processed in European sawmills, is related with the occurrence of knots [8]. These are the remains of branches that can be characterized by various shapes, dimensions, and different degrees of soundness. The presence of knots affects the mechanical properties of wood to a much greater extent than, for example, mold fungi. Studies indicate that only the sound, single knots with the diameter up to 10 mm are not decisive for assessing the quality of timber pieces. Moreover, their distribution along the length of wood tissue also plays a crucial role in the determination of timber quality [15,16,17]. Besides strength properties, knots affect the planning, sawing, gluing, finishing, and drying of wood. Therefore, they also negatively influence the properties of the manufactured beams [18]. Their occurrence has a particularly adverse effect when the knots are located in the bottom of the beam (tensile zone), and they are of less importance when they occur in the compression zone [19]. Because of this, most often knots are cut out of the timber, especially when they are rotten or running to the edge. As a consequence, this results in the production of a large number of wood pieces having limited, insufficient dimensions. The obtained elements are usually combined with the use of glue joints, such as finger joints. Many studies have been conducted regarding their geometry, effect on the mechanical properties, and ability to carry the load [20,21,22]. However, they are relatively difficult to execute; thus, there are still many ongoing studies aimed at finding the structurally simpler solutions for timber connecting, with or without the adhesive reinforcements [23].

A preferred solution for the advantageous use of wood in the manufacture of building elements is the production of glulam beams [24,25]. Hence, an attempt was made to develop new ways to obtain structural beams, with the use of non-full-size sawn timber pieces.

## 2. Materials and Methods

Based on a simple enquiry submitted to sawmills, annually converting approximately 15–20 thousand m^3^ pine timber for construction uses, it was found that sawn timber of 80 mm × 180 mm at the cross-section accounts for the greatest share of converted material (approximately 25%). In turn, timber of max. 9 m in length comprised of almost 50%, whereas over 50% within this group consisted of timber of approximately 6.5–7 m in length (Table 1). Some sawmills even report “*standard timber of max. 7 m*”. Information was collected directly from 10 sawmills in western Poland. In Poland, almost 90% of all sawmills are those converting up to 10 thousand m^3^ wood annually; thus, the number of sawmills with greater production capacity is very small, although they convert around 60% harvested large pine timber. Larger sawmills have better machinery, and they are equipped with more modern conversion lines; as a result, it is easier for them to adapt to changing demand and technological requirements.

**Figure 1:**
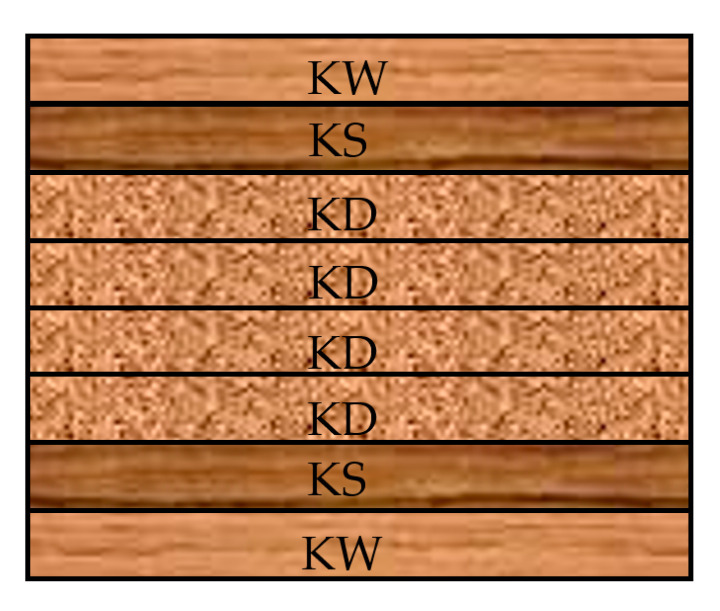
A diagram of timber layer arrangement for the TW variant.

**Figure 2:**
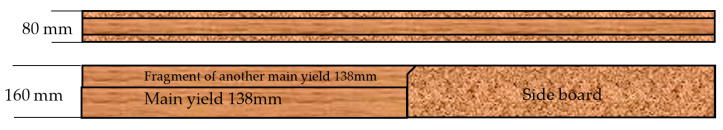
A diagram of timber layer arrangement for the ZS variant.

**Figure 3:**
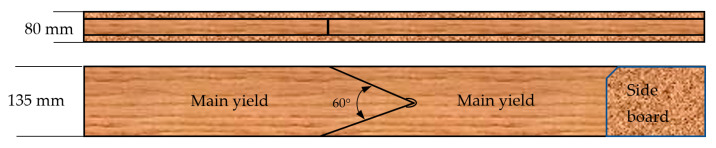
A diagram of timber layer arrangement for the KL variant.

However, regardless of the conversion method, the machine pool and automation of technological lines the conversion operations produce main yield obtained from the central sections of converted logs or boles and side boards from the circumferential sections. Side boards, typically of lesser thickness (20–28 mm), are characterised by their highly variable quality, depending on the log section, from which they were produced (butt end vs. top sections). This timber assortment is usually hardly marketable; due to its small thickness, it is not treated as structural timber. Another problem is connected with timber that is thick (over 28 mm) but relatively short, practically being a type of waste formed when converting timber material to meet customer specifications. Some companies use such timber to produce bonded panels (edge-glued, occasionally end-to-end glued) for sale to furniture manufacturers. Nevertheless, a preferable solution may be to use this timber as a structural material, since its sale is much more profitable, while additionally ensuring the more rational use of timber. This is because, after its lifecycle as structural timber, the material may be successfully used to manufacture furniture boards. In view of the above, it was decided to analyse the use of this pine sawn timber (*Pinus sylvestris* L.) to manufacture glulam beams. The following three variants were proposed:

(A) An eight-layer combined glulam beam (160 mm × 80 mm), manufactured from side boards of 20 mm in thickness and 80 mm in width (the TW variant, Figure 1). The timber was visually graded following the assumptions of PN-D-93021:2013-10 [26] and, on this basis, it was classified to four grades, i.e., KW (premium), KS (medium grade), KD (low grade), and out-of-grade. The beam was formed, so that the face layers were made from KW timber, the second and seventh layers from KS timber, and the core (four layers) consisted of KD timber.

(B) A three-layer beam (160 × 80), manufactured from two side boards of 21 mm in thickness and 160 mm in width and main yield boards of 40 mm in thickness and 138 mm in width (the ZS variant, Figure 2). Main yield was first bonded into boards approximately 97 cm in width, from which, lamellas of 160 mm in width and 40 mm in thickness were manufactured. The lamellas prepared in this way were then planed. Ultimately, their thickness was 38 mm.

(C) A three-layer beam (135 × 80), manufactured from two side boards of 21 mm in thickness and 135 mm in width and main yield boards of 38 mm in thickness and 135 mm in width (variant KL, Figure 3). Main yield boards were composed of two elements, wedge-jointed at a 60°angle, with the same MUF adhesive mixture applied for gluing the boards. In the timber section containing the wedge, prior to wedge cutting, a hole, 10 mm in diameter, was drilled at the recess tip.

Variant TW was meant to represent the use of narrow side boards, variant ZS—the use of narrow main yield boards; variant KL represented the use of short timber (in this case, less than 2 m).

While side boards with the length of 3500 mm were visually graded to assign them to individual grades, according to PN-D-93021:2013-10, it was also decided to determine the modulus of elasticity for each lamella in a four-point bending test, according to the methodology described by Mirski et al. [8]. In this way, the moduli of elasticity were established for timber elements used to manufacture beams KL and ZS.

The prepared sets, immediately before they were bonded to form beams, were machined to provide better quality of the surface to be glued.

**Figure 4:**
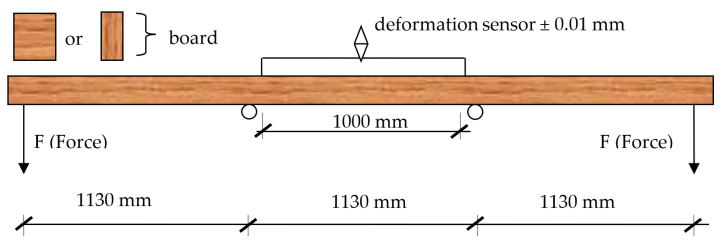
A scheme for bending strength test.

The beams were cold-pressed using MUF 1247 adhesive at 220–240 g/m^2^, mixed with the 2526 curing agent, added at 10% of dry resin mass. Both products are produced by Akzo Nobel. The mixture was prepared taking the conditions in the laboratory room into consideration. The adhesive was applied using a roller spreader. After the batch was fed to the press, the pressure of 0.48 MPa was applied. Beams were kept in the press for minimum 4 h and, after the conditioning period of approximately 2 weeks (temperature 20 ± 2 °C; relative humidity 65 ± 2%), they were tested for bending strength and modulus of elasticity in a four-point bending test, in accordance with the diagram shown in Figure 4. While constructing the test stand, the assumptions of the standard were used (EN 408:2013) [27]. The speed of movement of the loading head did not exceed 0.25 mm/s. The absorbed energy, according to the equation, was also determined (1) [28,29,30,31,32]:

(1) $$E_{e}=\int_{f1}^{f2}Fdf$$

where: *f*_1_ and *f*_2_ are the lower and upper integration limits for the deflection, respectively.

Twelve beams each were pressed for all the variants. The moisture content of each beam was recorded prior to the assessment of mechanical properties. Moisture content was determined using a HIT-3 resistive hygrometer.

Results obtained from direct measurements were analysed statistically using the Statistica ver. 13.0 package (Version 13.0, StatSoft Inc., Tulsa, OK, USA).

## 3. Results and Discussion

**Figure 5:**
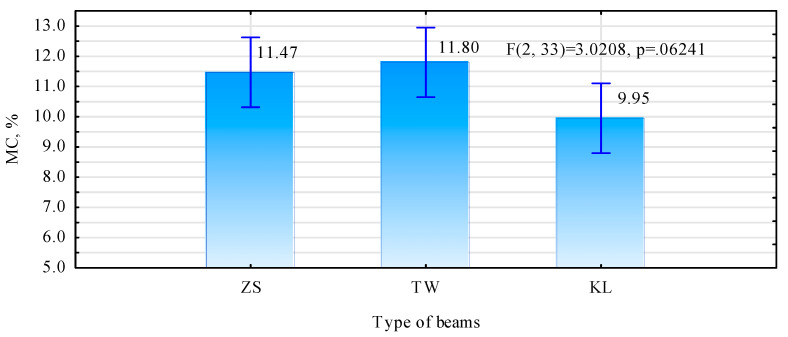
Moisture content of tested beams.

A significant characteristic of wood-based materials is connected with the dependence of their physical properties, particularly all the mechanical properties, on moisture content. The effect of moisture content on individual properties varies, with changes ranging from several to over a dozen percent. Among all the mechanical properties, the modulus of elasticity was most sensitive to changes in moisture content; according to different sources, the assumed range is from 1 to 4% change per 1% change in moisture content, within the range from 4% to 20% wood moisture content [33]. For this reason, the moisture content in each element was determined before measurements of bending strength and modulus of elasticity. As it results from the values presented in Figure 5, moisture content in the tested beam types varies slightly and ranges from 10% to 12%. However, there are no grounds for the rejection of the zero hypothesis, assuming identical moisture content in the analysed beam batches. Thus, it may be assumed that the evaluated beam types may be compared, without converting their mechanical properties to identical moisture content.

**Figure 6:**
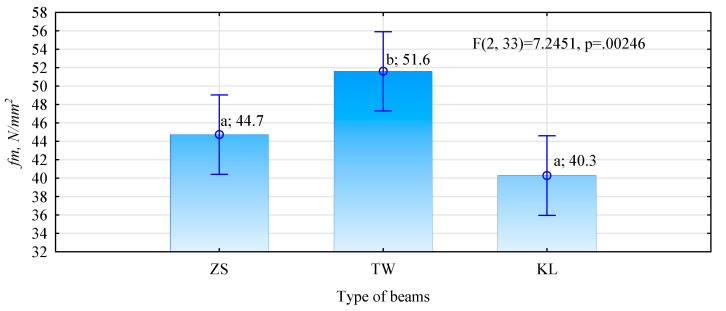
Bending strength in a 4-point test of manufactured beams (small letters denote results of the post-hoc LSD test).

Mean bending strength of such manufactured beams ranges from over 40 N/mm^2^ to almost 52 N/mm^2^ (Figure 6). The ANOVA results show that beams manufactured from eight side boards (the TW variant), i.e., evaluated in the horizontal arrangement of individual timber elements, are characterised by a much greater bending strength, compared to the other two types of beams. Moreover, while the difference was not statistically significant, the strength of beams manufactured using side boards bonded end-to-end using a wedge joint is slightly lower, compared to that of beams manufactured from edge-bonded main yield boards.

**Figure 7:**
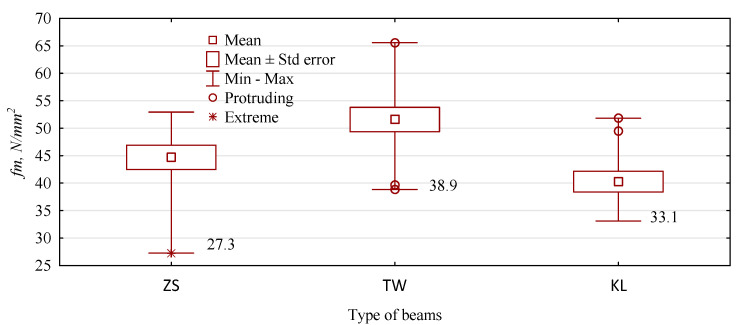
The distribution of static bending testing results and minimum values.

The grade of beams manufactured from glulam is determined by the characteristic value to a given property, rather than the mean recorded for a given group of beams. In the case of bending strength, it is the value of the fifth percentile, which, for the samples, is equal to the minimum value. In this case, beam-type ZS manufactured using edge-bonded main yield boards were the least advantageous variant (Figure 7). In turn, a relatively low variability was recorded for KL beams, i.e., those wedge-jointed in the core bonded end-to-end.

It may be assumed that the tested beam types meet the minimum requirements imposed on the proposed systems, i.e., at least GL 24c grade. This results from the fact that, according to the PN-B-03150:2000 standard [34], grade KW needs to meet the requirements for machine-graded timber grade C27, i.e., reach a minimum of 27 N/mm^2^ in the bending test. In all the beam types, the face layers were manufactured from this timber grade (KW). In view of the beam design/structure, the effect of core quality was considered to be less significant. As can be seen in data given in Table 2, the grade of the core in the case of three-layer beams was relatively high. In turn, in eight-layer beams low grade timber was placed in the beam core.

**Figure 8:**
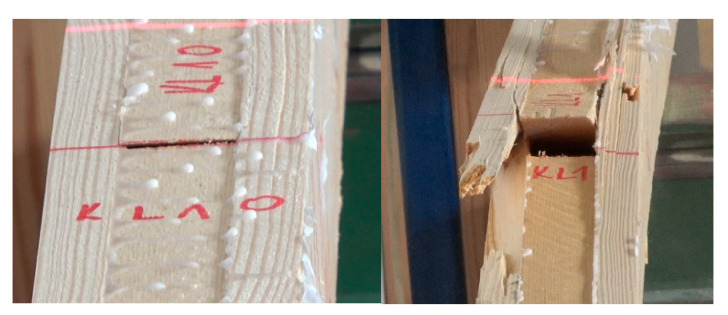
Failure in beam-type KL.

**Figure 9:**
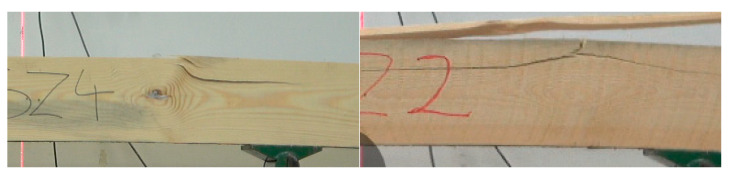
Examples of failure in beam-type ZS.

Considering values of the modulus of elasticity for timber used to manufacture KL and ZS beams lower strength values may have been expected for ZS rather than KL beams. However, tests showed that although the beam grade was determined by the lowest strength testing result, the discussed problem needs to be understood more broadly. In the case of beam-type ZS elimination of the weakest beam from the calculations results in the mean increasing by as little as 1.6 N/mm^2^, but the lowest value is as high as 36.8 N/mm^2^, compared to 35.8 N/mm^2^ for beam-type KL after the same calculation operation. Thus, the quality of both batches begins to be more uniform, even though beam-type ZS were manufactured from timber with a lower modulus of elasticity. It also needs to be stressed here that failure of beam-type KL is initiated at the wedge site (Figure 8 and Figure 9). In most cases, the main yield bonding site is split mainly in the zone subjected to tensile forces.

A significant characteristic of structural materials is connected with their stiffness or susceptibility to deflection under the applied force. In practice, the most common approach is to determine the modulus of elasticity for a given material, since it makes it possible to calculate other values related to the action of applied force. Moreover, the modulus of elasticity is a significant value affecting the grade of manufactured structural beams. The conducted statistical analysis showed that the mean values of the modulus of elasticity for the manufactured beam types range from 11.5 to 14.2 kN/mm^2^. The lowest value of the modulus of elasticity was recorded for beam-type ZS, while it was highest for beam-type KL. The obtained values differ statistically, which means that each batch is characterised by a significantly equal value of the modulus of elasticity. Within the range of the limits specified in the EN-14080:2013 [35] standard, the recorded values of the modulus of elasticity, except for beam-type TW, are consistent with the bending strength testing results, i.e., in view of these two parameters, the manufactured beams may be classified to the same grade. In the case of beams ZS, it would be grade GL24c, while for beam-type KL it would be grade GL32c. Thus, even the elimination of the low bending strength result for one beam from the ZS group would not change the grade of the manufactured batch, according to the respective standard. The situation is different in the case of beam-type TW, since they show much greater bending strength, which may be classified to grade GL32c, compared to the stiffness, which value defined by the modulus of elasticity classifies this beam type only to grade GL28c. For this reason, linking strength with the modulus of elasticity is justified only when searching for indexes, which may be safely used for structural systems.

However, it is also relatively important how the modulus of elasticity of timber is manifested in the modulus of elasticity of manufactured beams. A review of literature indicates that the stiffness of the glue line, for adhesives based on melamine or urea, tends to exceed that of wood; thus, it seems that the role of glue lines in manufactured beams, despite the presence of several glue lines, may be considered negligible. Thus, the recorded values of the modulus of elasticity are probably related with elasticity of timber.

**Figure 10:**
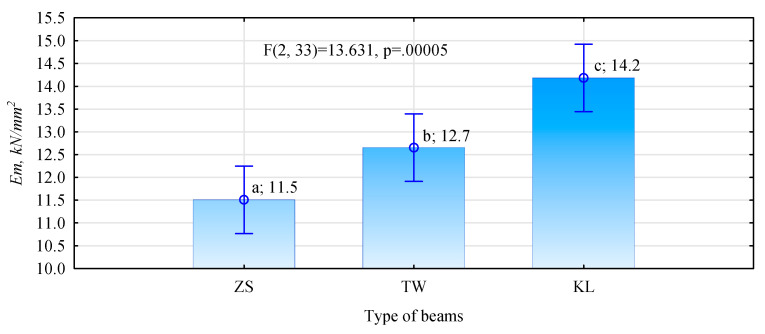
Modulus of elasticity of manufactured beams, specified in a four-point bending test (small letters denote results of the post-hoc LSD test).

Even based on the averaged values of the modulus of elasticity for the used timber, as presented in Table 2, the following values of the modulus of elasticity were expected, based on the substitute value [25,36]: for beam-type ZS, it was 13.3 kN/mm^2^; for type TW—11.3 kN/mm^2^; while 16.8 kN/mm^2^—for beam-type KL. Thus, as can be seen from values presented in Figure 10, in the case of beams, for which the modulus of elasticity was specified for timber in the bending test, the moduli of elasticity for beams are by approximately 13–15% lower than the expected value. An opposite situation was observed in the case of beam-type TW, which were graded visually. For this batch, the modulus of elasticity of beams was by approximately 13–15% higher than it would have resulted from the adopted assumptions. This may have been expected, since, typically, a grader when appraising a given specimen to be on the safe side tends to assign it to a lower grade.

**Figure 11:**
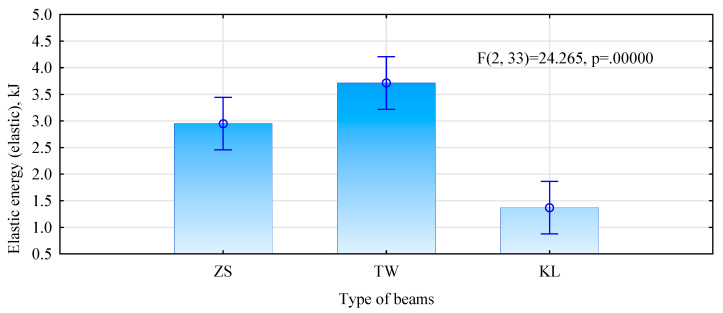
Elastic energy in the bending test.

**Figure 12:**
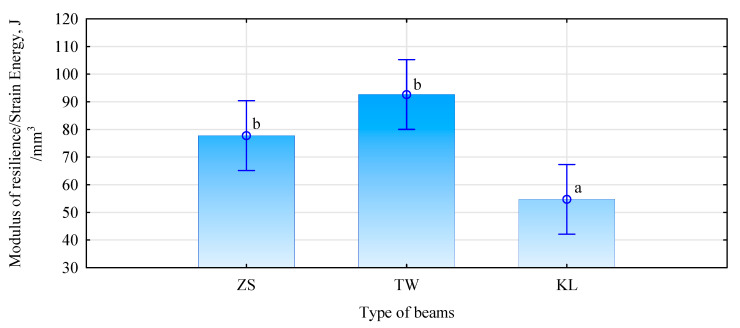
Elastic work in the bending test (small letters denote results of the post-hoc LSD test).

Significant indexes indicating the behaviour of elements in a bending test also include the values referring to the amount of energy required or absorbed (stored) in the analysed material before it fails or is permanently deformed. Figure 11 presents values of energy absorbed by the analysed systems, while Figure 12 gives work in the system within the elastic zone.

Both analysed physical values indicate a much lower potential to absorb energy by beam-type KL, compared to the other two types. This means that, despite high-elasticity, these beams lose their stiffness faster than is the case in the other beam types. This is probably caused by the development of microcracks or displacement of individual elements, in relation to one another, rather than the transition from the elastic to a plastic state. The results of this analysis, thus, confirm the observations related to the manner and location of failure in this beam type in the bending test.

## 4. Conclusions

It seems that the proposed and analysed beam models satisfy the requirements and needs of the market. Regardless of their design (the arrangement of layers and loads), all beam types show high mechanical properties, considerably exceeding the binding requirements for systems type GL24c. The highest strength, as well as elastic energy and elastic work in the bending test, are recorded for beams manufactured from visually-graded side boards. On the one hand, it may be connected with the better quality of timber used in this experiment, rather than the grades assigned. On the other hand, this beam’s arrangement of timber elements and glue lines was more advantageous than in the other models. Beam-type ZS was manufactured from edge-bonded main yield boards, cut into strips of a width—within which, two or three timber fragments may be found. In turn, beam-type KL has wedge joints to bind short elements. Although beam-types ZS and KL are manufactured using lower grade timber, they do not deviate considerably from beam-type TW, which is produced from lower grade timber—in this case, in the form of homogeneous lamellas. Essentially, although beam-type KL were characterised by the lowest mean bending strength and low values of work in the bending test, in view of the high modulus of elasticity, it was these beams that may be expected to meet the requirements for the highest grade specified in the EN 14,080 standard, i.e., grade GL 32c.

## Figures and Tables

**Table 1:** Conversion of round wood into structural timber.

Produced Cross-Section Variants				Available Lengths			
Dimensions (mm × mm)	Mean (%)	Min. (%)	Max. (%)	Dimensions (m)	Mean (%)	Min. (%)	Max. (%)
80 × 180	25	20	30	6.5–7	60	50	70
80 × 200	8	5	10	Up to 6	35	30	45
140 × 140	15	10	20	Up to 9	50	40	60
160 × 240	7	5	10	Up to 12	7	5	10
Others	52	35	60	Others	8	0	25

**Table 2:** Mean values of elastic properties of timber used in the experiments.

Beam Type	Layer		
	Face	Core	Face
	Mean Modulus of Elasticity, kN/mm^2^		
KL	16.9	16.7	16.8
ZS	13.3	13.0	13.3
TW	Assumed modulus of elasticity for individual grades		
	KW	KS	KD
	Modulus of elasticity, kN/mm^2^		
	11.5–12	10–11	9

## Data Availability

The data presented in this study are available on request from the corresponding author.
